# The Effects of Ultrasound on the Rehydration of Konjac Glucomannan/Soy Protein Isolate Gel and Simulation of Gas-Liquid Interface Evolution During the Rehydration Process

**DOI:** 10.3390/foods13244136

**Published:** 2024-12-20

**Authors:** Jiqiang Yan, Shizhong Jiang, Qin Wang, OuJun Dai, Zhuoer Yang, Biyao Huang, Ruoyu Huang, Zhenghao Chi, Yilan Sun, Jie Pang

**Affiliations:** 1 College of Computer and Information Sciences, Fujian Agriculture and Forestry University, Fuzhou 350002, China; yjq@fafu.edu.cn (J.Y.); mqhrygg@163.com (R.H.); chizhenghao@yeah.net (Z.C.); 2 College of Food Science, Fujian Agriculture and Forestry University, Fuzhou 350002, China; 18159933972@163.com (S.J.); d15158590879@163.com (O.D.); yangzhuoer2006@163.com (Z.Y.); 3 Cangzhou Strategic Reserves and Grain Oils Quality Inspection Center, Cangzhou 061000, China; hbsczs202206@163.com; 4 North Alabama International College of Engineering and Technology, Guizhou University, Guiyang 550025, China; biyaohuangcn@outlook.com

**Keywords:** konjac glucomannan, dry gels, rehydration, simulation, gel properties

## Abstract

Soy protein isolate (SPI) possesses potential gelling properties, making it suitable for gel-based applications. However, the gel network stability and mechanical properties of SPI are relatively poor and can be improved through modifications or by combining it with other polymers, such as Konjac Glucomannan (KGM). Combining SPI with KGM can overcome the poor gel network stability and mechanical properties of SPI, but it reduces the water-absorbing capacity of the gel network after drying, which affects the quality characteristics of plant-based protein rehydrated foods and limits the economic feasibility of soy protein foods. In this study, SPI and KGM are the main research objects. By using the alkali method to construct SPI/KGM dry gels with good gel properties, the influence of different ultrasonic powers on the rehydration kinetics and performance changes of SPI/KGM dry gels is examined. The speed and state of water entering the pores are simulated by constructing different pore-size capillary filling models, and the rehydration mechanism of the gel is elucidated. This study provides research ideas and a theoretical basis for the application of ultrasonic wave technology in the study of dry product rehydration performance.

## 1. Introduction

With rising economic prosperity and significant improvements in quality of life, health has become an increasingly prominent concern, particularly in the context of dietary choices. Consumers are now more discerning and informed about the food they consume, making dietary health a key focus in modern living. Plant-based foods, known for their natural purity, environmental sustainability, and safety, have steadily become a mainstream choice, earning widespread admiration and preference among a growing number of consumers [1].

Soy protein isolate (SPI)-based convenient ready-to-eat plant protein products, such as meat alternatives, are characterized by their rich nutritional value and favorable functional properties, positioning them as one of the predominant product types within the plant-based food sector [2,3]. However, during the processing of these products, both the freeze-drying and rehydration stages often face technical challenges that considerably hinder the rehydration performance of the food. Rehydration capacity is a key criterion for evaluating the quality of dehydrated foods and is typically assessed through comprehensive quantitative analysis, including rehydration rates, kinetics, and textural properties [4,5]. In the rehydration process of SPI-based hydrogels, the hydration characteristics of SPI, coupled with its dense network structure and the fine pore architecture of the dry gel, restrict the movement of moisture within the gel. This results in uneven water absorption, where the surface of the dry gel becomes moist while the interior remains dry [6]. Such uneven moisture distribution critically undermines the textural and sensory quality of the dry gel, thereby adversely affecting the mouthfeel experience of SPI-based foods [7]. Therefore, achieving uniform moisture absorption during the rehydration process—by modulating SPI’s hydration dynamics and enhancing the network pore structure of the dry gel—could significantly improve the overall quality of SPI-based products [8,9].

Polysaccharides exhibit remarkable biocompatibility, biodegradability, and renewability [10]. The hydroxyl (-OH) and amino (-NH_2_) groups within their molecular structure provide significant functionality due to their high chemical reactivity, making them indispensable in various applications, particularly in ready-to-eat convenience foods, where they enhance food quality and performance. Muhoza et al. [11] successfully integrated plant proteins with composite gelling technologies, significantly improving the functional properties of plant protein-polysaccharide gels while mitigating the adverse economic, environmental, and health impacts associated with animal protein [12]. Recent studies have shown that the incorporation of polysaccharides can notably enhance the moisture absorption properties of dried products. For example, the addition of guar gum improves the water resistance of freeze-dried dumplings [13], while incorporating carrageenan, konjac gum, and their combinations significantly accelerates the rehydration rate of Cantonese sausages [14]. Furthermore, the integration of polysaccharides has been shown to increase the moisture content in freeze-dried meatballs [15].

Recent research has also produced significant findings on the blending of konjac glucomannan (KGM) with SPI gels. Experiments on rheological properties have demonstrated that mixing KGM and SPI in specific ratios creates innovative gels with synergistic effects. When SPI solutions exhibit low viscoelasticity, the inclusion of KGM significantly enhances both the viscosity and elasticity of the mixture [16,17]. These composite gels, when used in the production of minced meat, improve texture while effectively retaining moisture and preventing loss during processing [18,19]. Thus, the combination of KGM and SPI not only facilitates the development of environmentally friendly functional materials but also enhances the hydrophilicity of SPI dry gels, improving their rehydration performance. Moreover, this approach contributes to the formation of a denser gel network, which strengthens the gel and holds promise for addressing challenges such as the collapse and dispersal of SPI dry gels upon rehydration [20,21].

Additionally, research has shown that factors such as material dispersion, pore size and quantity, the presence of air in the pores, and the hydrophilicity of interfaces can significantly impact water penetration during rehydration. Under consistent environmental conditions, an increase in the porosity of the porous medium correlates with a decrease in the time required for complete rehydration [22]. To optimize rehydration quality, researchers have primarily employed physical methods to adjust the pore structure of materials. Advanced techniques such as ultrasound [23,24,25], pulsed electric fields [26], and cold plasma [27,28], have been utilized for pre-treatment of samples, facilitating the formation of water-absorbing structures with appropriately sized pores and a loose, porous morphology.

Employing physical co-processing or impregnation methods [27] allows for the introduction of hydrophilic colloids into the minute pores of the molecular network. [27]. This strategy not only enhances the hydrogen bonding interactions between molecules but also further stabilizes the gel network while improving its water retention capacity. However, there is limited research focusing on post-treatment processes following drying to regulate the rehydration of dried products [29]. For instance, Wang et al. [30] demonstrated that ultrasound can enhance the rehydration rate of cured foods. Therefore, applying physical methods can improve the pore structure of SPI and dried gels, leading to the development of water-friendly architectures that enhance the rehydration performance of dried gels [31].

Challenges in the rehydration process of SPI were addressed by introducing KGM hydrophilic molecular chains to construct the SPI/KGM gel system. The performance of SPI/KGM dry gels during the ultrasonic rehydration process was thoroughly analyzed. Specifically, the influence of SPI/KGM on water flow, gel microstructure, and rehydration efficacy was investigated. A capillary filling model with varying pore sizes was employed to simulate the speed and state of water entering the pores. The rehydration mechanism of the SPI/KGM dry gel was elucidated and experimentally validated. This research provides a robust theoretical foundation for the rehydration properties of dry food gels, supporting the development of plant-based food products.

## 2. Materials and Methods

### 2.1. Materials

Konjac glucomannan was obtained from Hubei Yizhi Konjac Biotechnology Co., Ltd. (Yichang, China), while soy protein isolate was sourced from Nanjing Dulai Biotechnology Co., Ltd. (Nanjing, China). Anhydrous sodium carbonate, citric acid, and sodium bicarbonate were all purchased from Shanghai Maiklin Biochemical Technology Co., Ltd. (Shanghai, China).

### 2.2. Preparation of SPI/KGM Dry Gels

A 9% SPI solution was prepared by dispersing SPI in a 0.5% sodium carbonate solution and mechanically stirring at room temperature (25 °C) for 1 h. Then, 1% KGM powder was added to the SPI solution and stirred at 4192 g until fully mixed. The resulting sol was poured into silicone molds, with bubbles removed and the surface smoothed. The molds were then subjected to a water bath at 90 °C for 1 h. After natural cooling to room temperature, the gel was pre-frozen at −80 °C for 24 h. The KGM/SPI dry gel was then vacuum freeze-dried at −80 °C for 24 h and stored in a vacuum desiccator for future use.

### 2.3. Determination of the Rehydration Rate of SPI/KGM Dry Gels

The SPI/KGM dry gel was immersed in 150 mL of distilled water and subjected to ultrasonic treatment in a water bath at 40 °C. The ultrasonic treatment was performed at power levels of 0, 525 W, 600 W, 675 W, and 750 W using a water-bath ultrasonic system (manufacturer: Kunshan Ultrasonic Instrument Co. Ltd., Kunshan, China; type: KQ3200DE; frequency: 40 KHZ). The system employed a submerged ultrasonic probe where the target material was exposed to ultrasonic waves via the probe immersed in water. The ultrasonic waves were generated from a rod-shaped oscillator placed horizontally in the water bath, and the samples were irradiated from all directions during treatment to ensure uniform exposure.

Following the modified procedures described by Wang, Harnkarnsujarit, et al. [25,32], the gel samples obtained were treated under the specified ultrasonic conditions. Immediately after treatment, the samples were weighed to ensure data accuracy. Measurements were taken at intervals of 5, 10, 15, 20, 25, 30, 40, 50, 60, 80, and 120 min. At 180 min, the final weighing was conducted and recorded as the final rehydration mass. To ensure accuracy, each group of samples was tested in triplicate. The rehydration rate was calculated using the following formula:

(1) $$M_{C}=\frac{W_{t}-W_{0}}{W_{0}}$$

In this equation, $M_{C}$ [g] represents the rehydration rate of the dry gel at a certain moment, $W_{0}$ [g] is the initial mass of the dry gel before rehydration, and $W_{t}$ [g] is the mass of the freeze-dried gel after rehydration at a certain moment, respectively.

### 2.4. Construction of the Rehydration Kinetics Model for SPI/KGM Dry Gels

The rehydration process of dry products includes water absorption, swelling, and the leaching of soluble substances, which are three continuous processes [33]. Various phenomena such as molecular diffusion, convection, and capillary flow may occur. Due to the structural differences among various substances, the dominant mechanisms in the rehydration process also vary. The rehydration modeling employs a first-order kinetic model (2), the Peleg model (3), and the Weibull Equation (4).

The first-order kinetic model is as follows [34]:

(2) $$\frac{M-M_{e}}{M_{0}-M_{e}}=e^{-kt}$$

In this equation, M represents the rehydration rate at time t ($g\cdot g^{-1}$); *M_e_* is the equilibrium rehydration rate when t=∞($g\cdot g^{-1}$); *M*_0_ is the initial rehydration rate, expressed on a dry basis ($g\cdot g^{-1}$); and k is the rehydration rate constant (${min}^{-1}$).

Peleg’s model [35] is a two-parameter model used to describe moisture absorption curves. The equation is as follows:

(3) $$M=M_{0}+\frac{t}{K_{1}+K_{2}t}$$

In this equation, M denotes the rehydration rate at time t ($g\cdot g^{-1}$); $M_{0}$ is the initial rehydration rate ($g\cdot g^{-1}$); t represents the rehydration time (min); and $K_{1}$ is the Peleg rate constant (${min\cdot g}^{-1}\cdot g$).

The Weibull equation, as an empirical mathematical model, is widely used to describe the moisture distribution characteristics of dry materials during rehydration [36]. Its expression is as follows:

(4) $$\frac{M-M_{e}}{M_{0}-M_{e}}={e^{-}}^{(\frac{t}{a}{)}^{\beta }}$$

In this equation, M is the rehydration rate at time *t* ($g\cdot g^{-1}$); $M_{e}$ is the equilibrium rehydration rate ($g\cdot g^{-1}$); $M_{0}$ is the initial rehydration rate, expressed on a dry basis ($g\cdot g^{-1}$); *a* is the scale parameter; and *β* is the shape parameter.

### 2.5. Determination of Water-Holding Capacity of SPI/KGM Rehydrated Gels

The method for testing the water-holding capacity of the rehydrated gels was adapted from Zhang et al. [37]. Following complete rehydration under different testing conditions, the SPI/KGM rehydrated gels were weighed and recorded. The gels were then placed in 50-mL centrifuge tubes lined with flat gauze at the bottom to absorb moisture released during centrifugation. Centrifugation was performed at 16,768× *g* for 20 min. The water-holding capacity (%) was determined by measuring the sample weight before and after centrifugation, using the following formula:

(5) $$WHC=\frac{m_{1}}{m_{2}}\times 100\%$$

In this equation, $m_{1}$ represents the sample weight before centrifugation (g) and $m_{2}$ represents the sample weight after centrifugation (g).

### 2.6. SPI/KGM Rehydrated Gel Low-Field Nuclear Magnetic Resonance

Following the method described by Zhang et al. [38], the T2 relaxation times of the SPI/KGM rehydrated gel samples were measured at 32 °C using a MesoMR23-060H LF-NMR (Suzhou Nuumai Analytical Instrument Co., Ltd., Suzhou, Jiangsu, China) and the Carr-Purcell-Meiboom-Gill (CPMG) pulse sequence. The measurement parameters were as follows: sampling frequency of 200 kHz, repetition time of 10,000 ms, digital gain of 3, number of repetitions set to 3, data radius of 1, and 18,000 echoes with an echo time of 0.35 ms. The CPMG sequence was inverted, and the T2i (peak time) and M2i (area fraction) for each peak were recorded. Measurements were taken on SPI/KGM dry gel samples rehydrated for 3, 6, and 15 min under ultrasonic treatment at 675 W. For magnetic resonance imaging (MRI) analysis, a standard multi-layer spin echo (SE) sequence was used to capture two-dimensional proton density images during the rehydration process. The MRI parameters were set as follows: TE = 20.000 ms, TR = 500.00 ms, and averaging = 2.

### 2.7. Determination of Infrared and Secondary Structure of SPI/KGM Rehydrated Gels

SPI/KGM samples, freeze-dried after different rehydration treatments, were analyzed using an FTIR spectrometer. Approximately 1 mg of the dry gel sample was mixed with 99 mg of potassium bromide, ground using a mortar and pestle, and then compressed into a pellet with a thickness of 1–2 mm using a hydraulic press. The FTIR scans were conducted over a range of 4000 to 400 cm^−1^, with a resolution of 4 cm^−1^ and a total of 64 scans.

### 2.8. Determination of Color Differences in SPI/KGM Rehydrated Gels

The color of SPI/KGM gels rehydrated with ultrasonic assistance was measured using a colorimeter, calibrated with a white standard plate before use. The results were expressed in terms of L*, a*, and b* values. Each sample was measured six times to ensure color consistency [39].

### 2.9. Determination of the SEM Microstructure of SPI/KGM Rehydrated Gel

The microstructure of the SPI/KGM dry gel was analyzed using a scanning electron microscope (SEM). To enhance the visibility of the gel’s morphology, the samples were gold-sputtered in an argon atmosphere. SEM imaging was performed at an acceleration voltage of 10 kV and magnification of 200× to clearly display the structural features. The images were processed using Image J to determine the average pore diameter, with measurements repeated three times for accuracy.

### 2.10. Data Statistics and Analysis

The data from the two experimental groups were statistically analyzed using the IBM SPSS Statistics software (SPSSAU V23.0, SPSS Inc., Chicago, IL, USA) and Origin 2021b (OriginLab, Northampton, MA, USA). Each experiment was performed at least three times, and the results are expressed as the mean ± standard deviation. Analysis of variance (ANOVA) was conducted using SPSS, followed by Duncan’s multiple range test to evaluate group differences. A confidence level of 95% (*p* < 0.05) was considered statistically significant.

### 2.11. Gas-Liquid Interface Simulation for Rehydration Process Modeling

#### 2.11.1. Model Definition

**Figure 1:**
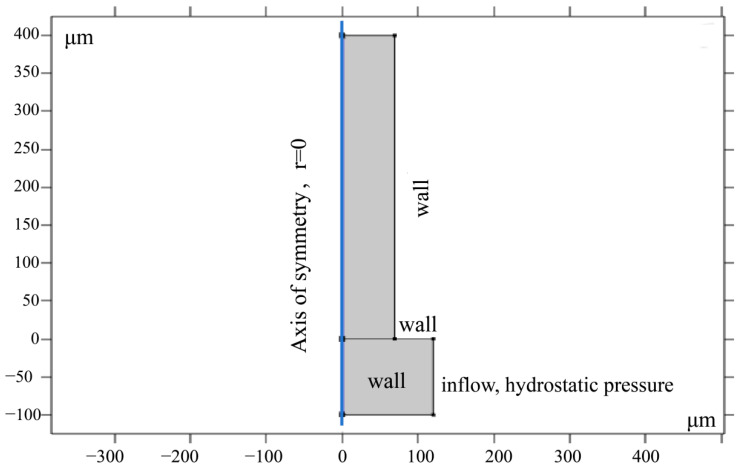
Axisymmetric geometry model for pore rehydration process.

To simplify the model and reduce computational complexity while maintaining high accuracy, axisymmetric geometry was used to construct the primary model (Figure 1). This model simulates narrow slit channels with pore diameters ranging from 50 to 500 μm, aiming to investigate the moisture adsorption behavior triggered by surface tension and the adhesion of the pore walls. Initially, these pores are completely filled with air. As the water column is positioned above the pores, capillary action gradually drives water into the small pores, causing the water level to rise.

During the rehydration process, contact angle data obtained from SPI/KGM dry gel experiments are used to explore the impact of varying contact angles on the adsorption behavior. The core of this model involves the use of level-set technology to precisely track the fluid interface. The capillary model consists of multiple cylindrical pore structures, each with a diameter between 50 and 500 μm. Each channel is designed with an independent reservoir, allowing water to flow in and out while maintaining a consistent water level.

At the start of the rehydration process, the pores are filled with air. Due to the adhesive properties of the pore walls, water begins to rise along the sidewalls. As the water level changes, surface tension develops at the air-water interface, causing rapid pressure fluctuations on both sides of the interface, which drives the water upward. The fluid level continues to rise until a stable state is achieved, where the gravitational force from the rising water height balances other forces, signaling the completion of the rehydration process.

#### 2.11.2. Level Set Method for Fluid Interface and Convection

The level set interface method is used to accurately construct interfaces for solving convection equations. The level set function, denoted as φ, represents the interface, with its 0.5 contour characterizing the interface’s position. Specifically, φ is set to zero in air and one in water, meaning φ can be interpreted as the volume fraction of water at any given point. This allows for the description and tracking of the dynamic migration of the two-phase fluid interface. The behavior of the interface is governed by the following mathematical formulation:

(6) $$\frac{\partial \varphi }{\partial t}+u\cdot \Delta \varphi =\gamma \Delta (\varepsilon \Delta \varphi -\varphi (1-\varphi )\frac{\Delta \varphi }{\left|\Delta \left.\varphi \right|\right.})$$

In the above equation, the parameter represents the thickness of the interface. For numerical stability and to ensure the accuracy of simulation results, the interface thickness parameter is typically set as ε=hc/2, where hc denotes the characteristic grid size of the interface region. The parameter γ is used to regulate the frequency of model reinitializations, with its optimal value corresponding to the maximum flow velocity observed in the model.

To describe the multiphysics coupling characteristics, the density and viscosity are defined using the following equations:

(7) $$\rho =\rho_{\mathrm{air}}+(\rho_{\mathrm{water}}+\rho_{air})\varphi$$

(8) $$\mu =\mu_{\mathrm{air}}+(\mu_{water}-\mu_{air})\varphi$$

According to the definitions in the equation, the density and viscosity coefficients vary smoothly across the fluid interface. The function δ can be approximated as follows:

(9) $$\delta =6\left|\varphi (1-\varphi )\right|\left|\Delta \varphi \right|$$

The interface normal can be calculated using the following equation:

(10) $$n=\frac{\Delta \varphi }{\left|\Delta \varphi \right|}$$

#### 2.11.3. Mass and Momentum Transfer

The model employs the Navier-Stokes equations to simulate mass and momentum transfer mechanisms within an incompressible fluid system. To more accurately capture the complexity of fluid behavior, the effects of surface tension are incorporated as a critical factor. Accordingly, the Navier-Stokes equations are extended to describe and predict fluid behavior under the influence of surface tension, as represented by the following equation:

(11) $$\rho \frac{\partial u}{\partial t}+\rho (u\cdot \Delta )u=\Delta \cdot \left[-pI+\mu (\Delta u+\Delta u{)}^{T}\right]+F_{st}+\rho g$$

(12) Δ⋅u=0

where ρ is the density (kg/m^3^), μ is the dynamic viscosity (Ns/m^2^), u is the viscosity (m/s), p is the pressure (Pa), g is the gravitational vector (m/s^2^), and $F_{st}$ is the surface tension acting at the air-water interface.

#### 2.11.4. Surface Tension

In the “level set” interface, the formula for calculating surface tension is

(13) $$F_{\mathrm{st}}=\sigma \delta \kappa n$$

where *n* is the normal to the interface, σ is the surface tension coefficient (N/m), δ is the Dirac delta function, and k=−∆·n is the curvature.

#### 2.11.5. Initial Conditions

In the initial stage of the simulation, the model’s water reservoir is filled with water, while the capillary channel above is completely filled with air, and the initial velocity is zero.

#### 2.11.6. Boundary Conditions

Inlet: To simulate the pressure at the inflow boundary, the hydrostatic pressure is set as p=ρgz. The pressure boundary condition automatically accounts for hydrostatic pressure; therefore, the hydrostatic pressure at the inlet is set to 0. This boundary permits only water to enter, with the water volume fraction specified as 1.

Outlet: The pressure at the outlet is also set to 0, as there is no pressure influence from the upstream inflow boundary. This boundary functions as an outflow boundary, requiring no additional conditions for the level set function.

Wall: The “wetting wall” represents the contact interface between the fluid and the solid surface. In the level set method, it captures the coupling characteristics of multiphysics. The velocity component normal to the wall is set to 0, expressed as:

(14) $$u\cdot n_{wall}=0$$

In addition, this model also considers the friction boundary force, which is calculated using the following formula:

(15) $$F_{fr}=-\frac{\mu }{\beta }u$$

where: β represents the slip length.

## 3. Results

### 3.1. Rehydration Properties and Water Retention of SPI/KGM Hydrogels

**Figure 2:**
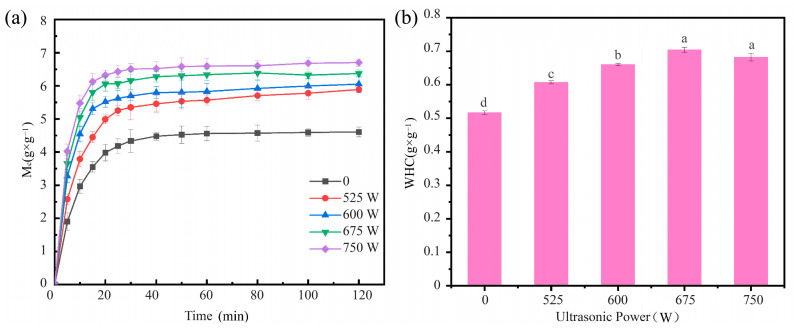
SPI/KGM Dry gel rehydration curves and water retention images. (**a**) Rehydration curves of SPI/KGM dry gel at different ultrasonic power levels. The lines represent rehydration at different ultrasonic power levels (0 W, 525 W, 600 W, 675 W, 750 W). (**b**) Effect of Ultrasonic Power on Water-Holding Capacity (WHC) of SPI/KGM hydrogels. Different letters above bars indicate significant differences (*p* < 0.05).

The moisture content of SPI/KGM dry hydrogels increases progressively with both the duration of rehydration and the ultrasonic power applied. As illustrated in Figure 2a, at the 20-min mark, the rehydration rate of the ultrasonic-treated samples shows a marked enhancement compared to the control group. Notably, the sample subjected to 675 W ultrasonic treatment achieves the highest ultimate rehydration rate of 6.52 g/g. After 40 min, all samples gradually approach equilibrium in moisture content. When extending the rehydration duration to 120 min, the rehydration rates of the samples treated at ultrasonic powers of 525 W to 675 W increase by approximately 27.79%, 31.35%, 45.41%, and 38.25%, respectively, compared to the control group. These findings clearly demonstrate that ultrasonic treatment significantly shortens the rehydration time of SPI/KGM hydrogels while effectively enhancing their equilibrium rehydration rate [40].

Figure 2b illustrates the effect of ultrasonic power on the water-holding capacity (WHC) of SPI/KGM hydrogels. Compared to the control group, ultrasonic treatment significantly enhanced the WHC of SPI/KGM hydrogels. As the ultrasonic power increased from 0 to 675 W, the water-holding capacity of rehydrated SPI/KGM gels progressively improved. However, further increases in ultrasonic power beyond 675 W led to a decline in WHC. Specifically, at 675 W, the WHC improved by approximately 37.25% compared to the control group (*p* < 0.05). This suggests that the ultrasonic-assisted rehydration process enhances the water retention capacity of SPI/KGM gels, likely due to the cavitation effects induced by ultrasound. Cavitation generates localized high-pressure and temperature conditions within the solid matrix, significantly altering the structure and properties of both SPI and KGM. In the case of SPI, the shear forces generated by cavitation, combined with the high temperature and pressure, lead to the denaturation of protein molecules. This disruption of the protein’s three-dimensional structure results in altered hydrogen bonding, ionic interactions, and hydrophobic interactions, which expose the protein’s active sites and enhance its gelation ability. Furthermore, cavitation facilitates the aggregation of SPI molecules, allowing them to form a more stable network structure, which improves the mechanical strength and stability of the gel. These structural changes also enhance the hydration of SPI, increasing its ability to adsorb and retain water, thereby improving the overall water retention and gel properties. Similarly, for KGM, cavitation causes molecular degradation and cross-linking. The ultrasonic energy breaks down the long-chain structure of KGM, creating shorter fragments that more readily combine with other molecules to form a stable gel network. Additionally, cavitation promotes cross-linking between polysaccharide molecules, further strengthening the gel’s mechanical properties and enhancing its thermal stability. The cavitation effect also improves the water retention and rheological properties of KGM, which is particularly beneficial in food processing applications, as it contributes to better texture and extended shelf life. These findings highlight the significant role of cavitation in improving both the water retention and structural properties of SPI/KGM hydrogels, making them more suitable for various food applications [41,42].

### 3.2. SPI/KGM Gel Rehydration Kinetic Model Construction

In this study, several rate models—the First-Order Kinetic model, Peleg model, and Weibull model—were employed to estimate the rehydration rate of SPI/KGM hydrogels. Although simulations were conducted using experimental data, the use of well-established rate models allows for comparative analysis and a better understanding of the underlying rehydration kinetics. These models have been widely applied in similar systems and provide a reference point for evaluating the complexity of the rehydration process.

Each model brings distinct advantages, reflecting different facets of the rehydration process. The First-Order Kinetic model, commonly used for exponential processes, was considered as a basic benchmark, although it did not adequately fit the data. The Peleg model, offering more flexibility, was tested but also showed limitations under higher ultrasonic powers. The Weibull model, known for its versatility in time-dependent processes, provided the best fit in this study, offering both statistical robustness and physical relevance.

#### 3.2.1. First-Order Kinetic Model

**Figure 3:**
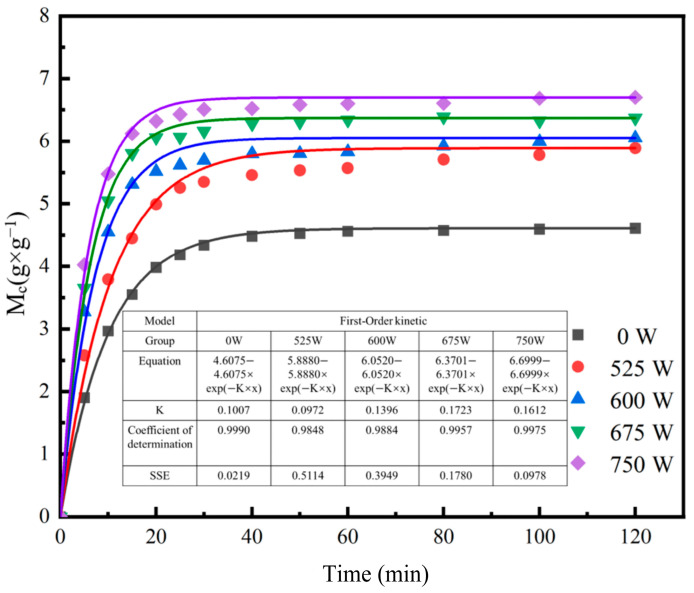
Model fit of SPI/KGM gel rehydration data to the first-order kinetic model.

In the first-order kinetic model, the constant K represents the rehydration rate, with an increase in K corresponding to a higher rehydration speed [43]. As shown in Figure 3, the value of K fluctuates with increasing ultrasonic power, first rising, then decreasing, and rising again. However, the model’s parameters, R^2^ and SSE, are not ideal, indicating a considerable discrepancy between the predicted and experimental data. This suggests that the first-order kinetic model does not provide an accurate fit for the rehydration process of SPI/KGM dry gel and is therefore unsuitable for effectively modeling this process [44].

#### 3.2.2. Peleg Model

**Figure 4:**
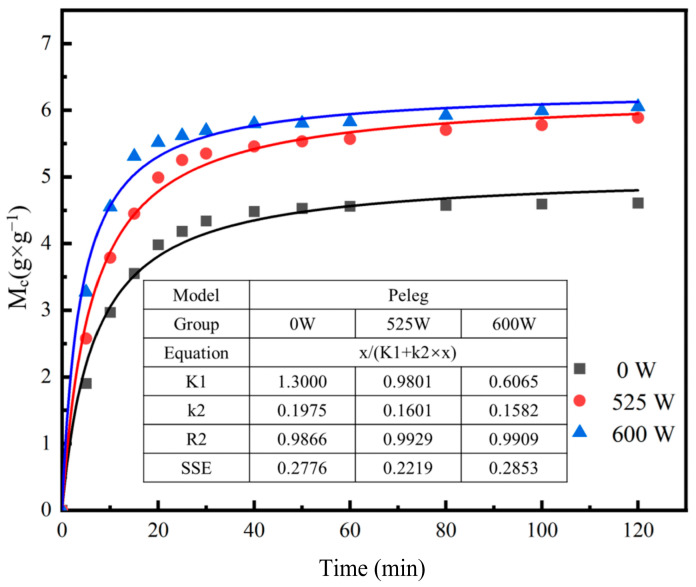
Fitting of SPI/KGM gel rehydration data to the Peleg model.

As shown in Figure 4, with the gradual increase in ultrasonic power, the values of K1 and K2 consistently decrease. At the same time, the equilibrium rehydration rate of the product increases. However, when the ultrasonic power exceeds 600 W, the model’s fit fails to converge. During the analysis of the SPI/KGM rehydration gel data, it was observed that the Peleg model exhibited relatively low accuracy during the fitting process, suggesting that it is not fully suitable for predicting the rehydration behavior of this dried product [45].

#### 3.2.3. Weibull Model

**Figure 5:**
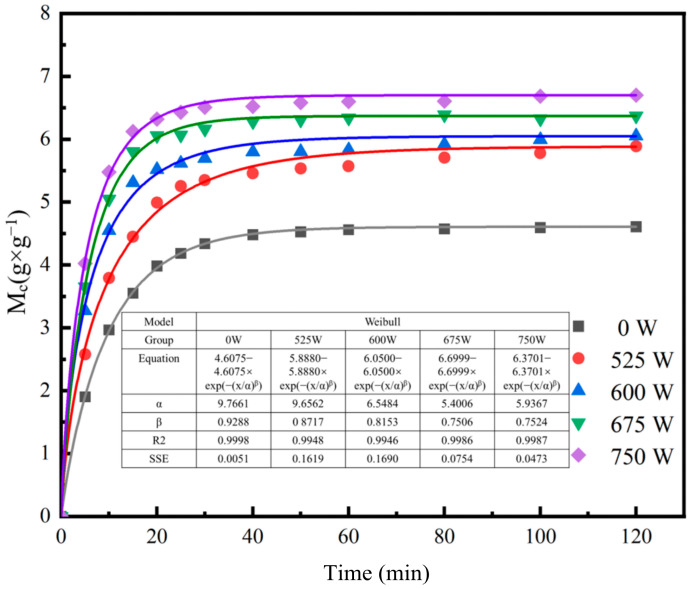
Fitting of SPI/KGM gel rehydration data to the Weibull model.

The rehydration rate data for each time period was fitted to the Weibull model to obtain the fitting parameters α and β. As shown in Figure 5, during the ultrasonic rehydration process, the α value decreases gradually with increasing ultrasonic power, while the β value initially decreases and then increases. The rise in β is likely attributable to excessive structural damage caused by higher ultrasonic power to the SPI/KGM rehydration gel. Consequently, as ultrasonic power increases, the rehydration rate of SPI/KGM improves, leading to a shorter time required to reach 63% rehydration [34].

A comparison of the R^2^ and SSE values between the Weibull model and the other two models suggests that the Weibull model provides a better fit to the data in this study. It shows the lowest SSE value and the highest R^2^ (≥0.9946). However, it is important to note that model selection should not be based solely on statistical metrics such as R^2^ and SSE. The physical relevance of the model to the rehydration process must also be considered. In this case, the Weibull model’s ability to capture the key features of the rehydration process aligns with its statistical performance, making it a suitable choice for describing the SPI/KGM rehydration gel under the various rehydration conditions.

### 3.3. SPI/KGM Rehydration Gel Low-Field Nuclear Magnetic Resonance Analysis

**Figure 6:**
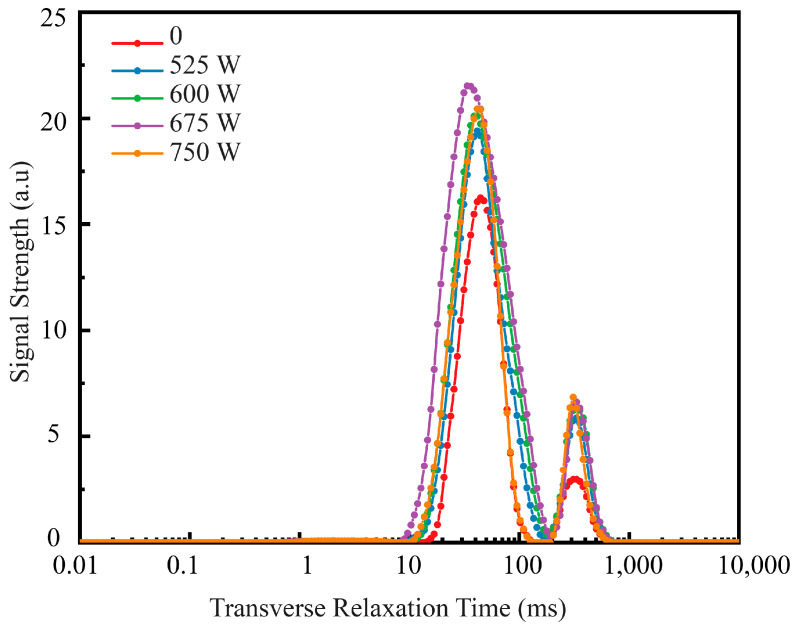
Low-field NMR relaxation time distribution of SPI/KGM dry gel under different ultrasonic power levels.

In Figure 6, the LF-NMR decay curve that conforms to the T2 distribution shows three peaks, corresponding to bound water (T21, 0.64–6.87 ms), non-flowing water (T21, 8.40–204.91 ms), and free water (T22, 204.91–714.94 ms) [46]. The peak areas A21, A22, and A23 represent the moisture content associated with the three different water distributions described above. It is evident that the SPI/KGM rehydration gel predominantly consists of non-flowing water. To analyze the data, T2 values from low-field NMR relaxation images and the signals corresponding to different ultrasonic treatments were used to determine the peak areas A21, A22, and A23, as shown in Table 1. As ultrasonic power increases, the T21 relaxation time exhibits a rising trend, reflecting a shift from free-flowing water to bound and non-flowing water in the SPI/KGM rehydration gel system. This trend is indicative of the conversion of free-flowing water into more tightly bound forms due to the effects of ultrasound. Notably, the peak area and peak value of the rehydration gel treated with ultrasound are significantly greater than those of the control group. When ultrasonic power reaches 675 W, the peak area A22 attains its maximum value of 1627.3083, signaling a profound alteration in the gel structure. Concurrently, the T22 relaxation time following ultrasonic treatment is the shortest in the entire system, at 33.70 ms, further underscoring the ability of ultrasonic treatment to effectively modulate the gel’s internal moisture state.

**Figure 7:**
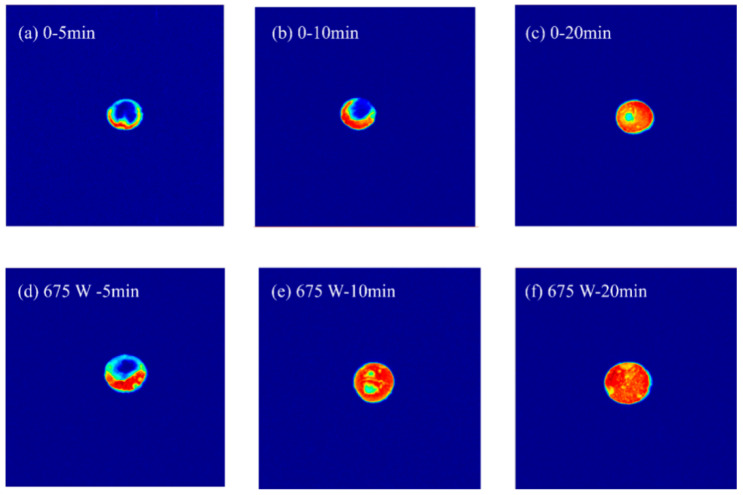
Moisture migration under ultrasonic rehydration conditions.

The effect of ultrasonic rehydration on the moisture distribution of the SPI/KGM dry gel is shown in Figure 7. It was observed that the brightness of the rehydrated SPI/KGM gel samples decreased progressively from the outer surface to the center, indicating that moisture migrated from the periphery toward the core of the gel. After 5 min of rehydration, the proton density in the ultrasonic group was higher than in the non-ultrasonic group, suggesting that ultrasound enhances the initial absorption rate of the gel. After 10 min of rehydration, the color distribution in the ultrasonic group primarily appeared red, indicating a higher rehydration rate and nearing the maximum rehydration capacity. Compared to traditional rehydration methods, ultrasonic treatment demonstrated a faster rehydration speed, significantly reducing the overall rehydration time [47].

### 3.4. Infrared and Secondary Structure of the SPI/KGM Rehydrated Gel

**Figure 8:**
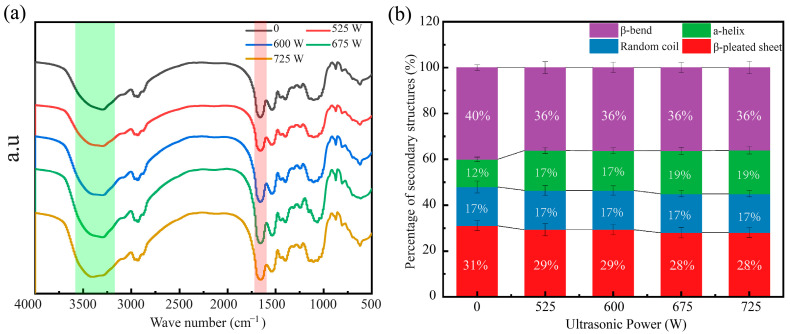
SPI/KGM infrared spectra and secondary structure impact diagram. (**a**) Fourier transform infrared spectrum of the SPI/KGM ultrasonic rehydrated gel, with different colors representing varying ultrasonic power levels (0 W: black, 525 W: red, 600 W: blue, 675 W: green, 725 W: yellow). (**b**) Effect of ultrasonic treatment on the secondary structure of the SPI/KGM gel, where purple indicates β-bend, green indicates α-helix, blue indicates random coil, and red indicates β-pleated sheet.

Freeze-dried gels subjected to ultrasonic rehydration at 0 W, 525 W, 600 W, 675 W, and 750 W were analyzed using FTIR spectroscopy. Figure 8a presents the FTIR spectra of SPI/KGM rehydrated gel samples treated with different ultrasonic powers. Compared to the spectrum of the untreated SPI/KGM gel, no new absorption peaks were observed in the gels treated with ultrasound. This indicates that ultrasonic rehydration is a physical process rather than a chemical one. All SPI/KGM rehydrated gels exhibited a significant absorption peak around 3300 cm^−1^, corresponding to -OH stretching vibrations, which reflects hydrogen bonding interactions within the gel system [48].

Ultrasonic treatment is known to induce physical effects such as cavitation, shear forces, and thermal effects, which can impact the protein’s secondary structure. Cavitation, in particular, results in the formation and collapse of microbubbles in the solution, creating high shear forces that can disrupt the spatial arrangement of protein molecules. These forces can lead to changes in hydrogen bonding patterns and the molecular conformation of proteins. The shear forces also lead to partial denaturation of proteins, which could alter the stability of secondary structural elements like α-helices, β-sheets, and random coils. Additionally, the thermal effects from ultrasonic energy absorption can influence the protein folding and the dynamics of molecular interactions, further contributing to structural changes.

Analysis of the amide I band (1600–1700 cm^−1^) in the red region of the FTIR spectrum revealed changes in the secondary structure of the protein. The amide I band comprises components corresponding to α-helices (1650–1660 cm^−1^), random coils (1660–1665 cm^−1^), β-sheets (1665–1670 cm^−1^), and β-turns (1670–1680 cm^−1^). Baseline correction, deconvolution, and second derivative fitting of the FTIR spectra for the ultrasonically treated gels minimized residuals, enabling statistical determination of the proportion of each structural component.

As shown in Figure 8b, the secondary structure of the SPI/KGM rehydrated gel is predominantly composed of β-sheets and β-turns. Ultrasonic treatment induced noticeable changes in the protein’s secondary structure. As ultrasonic power increased, α-helix content also increased, likely due to enhanced hydrogen bonding within the gel during rehydration. This increase in α-helix formation can be attributed to the energy from ultrasonic waves overcoming the energy barrier required for the formation of these more stable structural elements. Conversely, the β-sheet content decreased, which may be attributed to ultrasonic effects disrupting the spatial arrangement of protein molecules, breaking the layered structure of β-sheets, and leading to a shift towards more flexible structures like random coils or α-helices. The content of random coils remained relatively unchanged, while variations in β-turn content were minimal.

### 3.5. Color Differences in the SPI/KGM Rehydrated Gel

From Table 2, it is evident that ultrasonic treatment induces notable changes in the color of rehydrated SPI/KGM. The L* value of SPI/KGM after ultrasonic rehydration first increases and then decreases, while the a* (red-green) and b* (yellow-blue) values show a decreasing trend. The variation in L* values reflects the rehydration rate of the samples after the rehydration process [49]. In this study, the L* value of the rehydrated gel at an ultrasonic power of 675 W increased by approximately 7.1% compared to the non-ultrasonic group.

The cavitation effect induced by ultrasonic treatment plays a crucial role in pigment loss and color degradation. During cavitation, rapid bubble formation and collapse occur, creating localized high-pressure and high-temperature conditions. These extreme conditions can cause the molecular structure of pigments to break down, leading to their degradation and oxidation. As a result, the color intensity of the gel decreases, particularly affecting the a* (red-green) and b* (yellow-blue) values, which reflects a reduction in vibrancy. The cavitation-induced color change is thus primarily attributed to the mechanical disruption of the pigment molecules, coupled with the thermal effects of ultrasonic treatment.

The process of protein rehydration also contributes to color changes, independent of ultrasound treatment. Rehydration increases the water content of the gel, which can influence its color by altering its light-scattering properties. As the water content increases, the gel becomes more transparent, which can lead to a dilution of the color intensity, further contributing to the decrease in a* and b* values. This effect highlights the interplay between water content and ultrasonic treatment in determining the overall visual appearance of the rehydrated gel.

### 3.6. SEM Results of the Effect of Ultrasound on the SPI/KGM Rehydrated Gel

**Figure 9:**
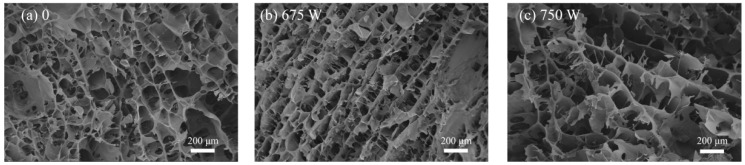
Microstructure of SPI/KGM gel after sonication.

As illustrated in Table 3 and Figure 9, the microstructure of the SPI/KGM rehydrated gel features a loose, porous structure with irregularly sized and unevenly distributed pores. In contrast, the traditionally rehydrated gel displays a less uniform pore distribution, with some regions being flat and devoid of pores. The gel treated with ultrasound at 675 W shows finer pores, likely due to the cavitation effect, which creates microchannels within the SPI/KGM matrix, increasing its porosity. However, when ultrasonic power is increased to 750 W, the gel exhibits larger pores, suggesting that excessive ultrasonic energy may damage the sample, leading to the enlargement of the pores. These microstructural variations in the SPI/KGM rehydrated gel are closely linked to water molecule flow from the surface to the interior, as well as its inherent water absorption capacity. The pore structure facilitates faster inward flow of water. Ultrasound treatment generates significant pressure gradients within the sample, causing water molecules in the SPI/KGM rehydrated gel to move more rapidly, which not only accelerates the rehydration process but also induces structural changes, forming new channels that enhance the rate of rehydration transfer [50].

### 3.7. Experimental Validation and Analysis of Gas-Liquid Interface Evolution

#### 3.7.1. Main Rehydration Process

**Figure 10:**
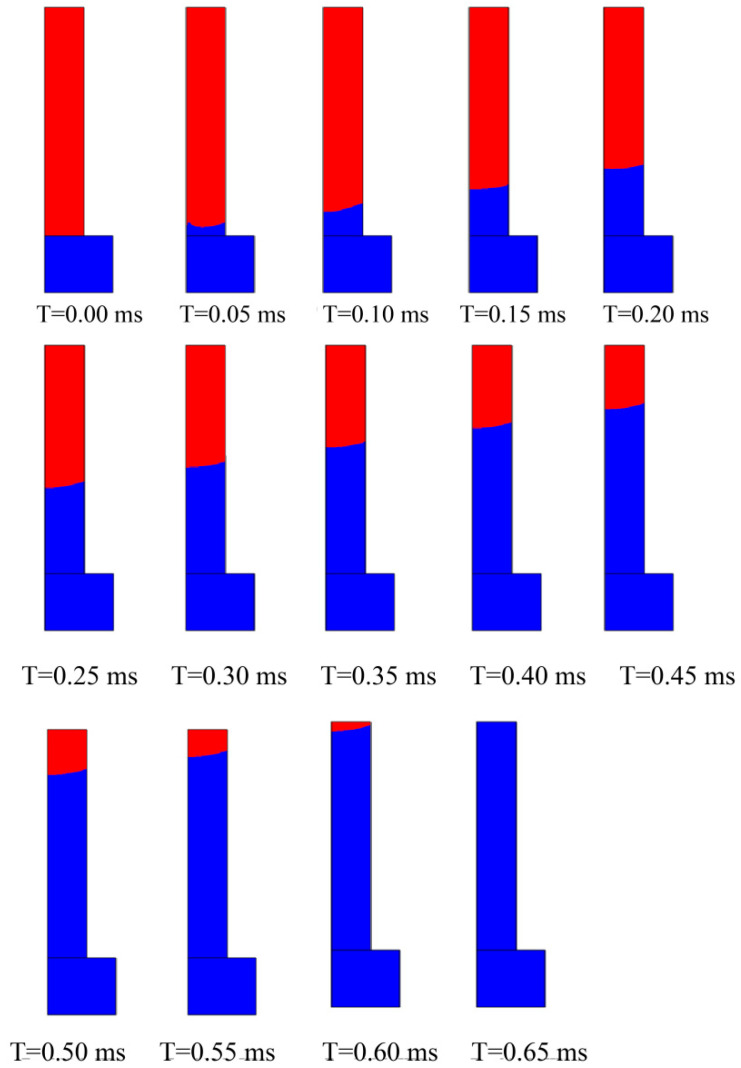
Interface location within 0.65 ms for a pore with radius R1. (Blue represents water, red represents dry gel.)

Computer simulations effectively capture minute fluctuations over short time intervals. Figure 10 depicts a vertical hole with a radius of 69.11 μm and a length of 400 μm positioned on a static water surface. After a period of water adsorption, interface data were recorded at various time points to provide detailed insights into the evolution of the gas-liquid interface during the rehydration process.

At the initial stage of the liquid interface formation, the water exhibited a concave upward surface. During this phase, the amplitude of interface fluctuations gradually increased, and the contact angle approached the range defined by the model. Once the contact angle aligned with the specified parameters, surface tension generated by the curvature of the liquid interface caused water to rise vertically within the tube. Throughout the process, the model’s instantaneous activation induced slight vibrations along the horizontal plane. At 0.65 ms, the hole was fully covered with water.

#### 3.7.2. Evolution Simulation Results with Pore Diameter as the Variable

**Figure 11:**
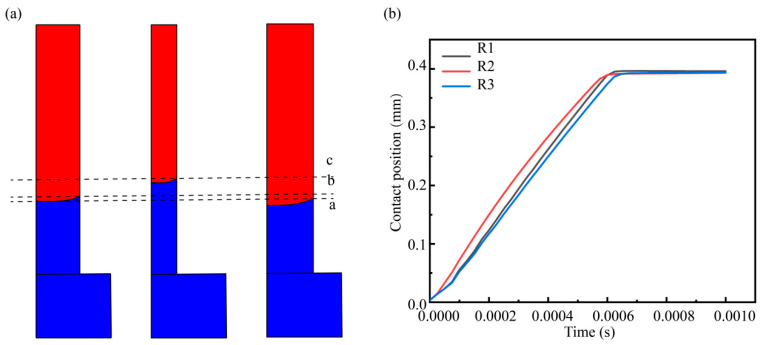
Simulation results showing the evolution of the interface with pore size as a variable. (**a**) Interface position for different pore size models at T = 0.2 ms. (**b**) Temporal variation of the interface/wall contact point position for different pore size definitions. (Blue represents water, red represents dry gel.)

The internal spatial structure of the gel plays a crucial role in determining rehydration efficiency. Ultrasonic treatment induces the formation of pore structures in SPI/KGM dry gels, varying in diameter, arrangement, orientation, and density. To simplify the complex model, these structures are approximated as vertical pores with a length of 400 μm for the purposes of this study. Using the primary pore diameters identified at different ultrasonic power levels in Section 3.6, the radius R of the cylindrical pores in the model is set to half of the main pore diameter, calculated as shown in Table 4:

To analyze the effects of different pore diameters on rehydration speed, pore models with varying radii were constructed based on the definitions provided in Table 3. The changes in the position of the interface/wall contact point over time for each pore diameter were observed, yielding the results shown in Figure 11a. Comparison with the curves of the contact angle model indicates that pore diameter significantly influences the water surface rising speed, as illustrated in Figure 11b. Notably, as the pore diameter decreases, the rehydration speed exhibits a clear increasing trend.

**Figure 12:**
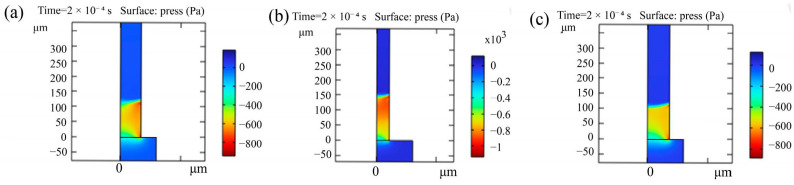
Internal pressures of different aperture models at T = 0.2 ms. (**a**) internal pressure with radius 69.11, (**b**) internal pressure with radius 41.67, and (**c**) internal pressure with radius 73.39.

Figure 12 illustrates the pressure distribution at the fluid surface at T = 0.2 ms. The figure reveals pressure jumps of varying magnitudes across the fluid surface, ranging from approximately 200 Pa to 1200 Pa. Notably, as the pore diameter decreases, the pressure jumps within the pores tend to increase. This pressure variation is primarily attributed to surface tension, which facilitates the upward movement of both water and air along the vertical pipe. Fauster et al. [23] highlighted that factors such as the shape, surface roughness, and porosity of the physical medium can all influence the simulation results. The pore diameter size directly affects the rate of moisture transfer through the pores. The simulation findings further indicate that smaller pore diameters accelerate the wetting process, resulting in faster rehydration speeds at the interface.

## 4. Discussion

The rehydration curve data were analyzed using the first-order kinetic equation, Peleg model, and Weibull model. Among these, the Weibull model exhibited the highest accuracy for describing the rehydration process of SPI/KGM rehydrogels, with R^2^ values exceeding 0.9946 and reaching up to 0.9998. This confirms the model’s superiority in predicting rehydration performance and highlights the effectiveness of ultrasound-assisted rehydration in enhancing both rehydration rates and water-holding capacity.

Under 675 W ultrasonic treatment, the rehydration rate of the SPI/KGM rehydrogel increased by 45.41% compared to the non-ultrasonic group, while water-holding capacity reached 70.48%. LF-NMR analysis showed that non-flowing water dominated the gel, with free water progressively converting to bound or non-flowing water due to ultrasonic cavitation within the gel matrix, facilitating the retention of strongly attached water. Pseudo-color imaging confirmed the highest proton density in the 675 W sonication group, aligning with the observed water-holding capacity.

FTIR analysis revealed significant secondary structure changes in the gel under 675 W ultrasound, with a shift from predominantly β-sheets and β-turns to α-helices. This structural transition is indicative of protein denaturation, where high shear forces, cavitation effects, and localized temperature changes break hydrogen bonds, disrupt hydrophobic interactions, and lead to partial disulfide bond cleavage. These effects promote protein chain flexibility, allowing for more effective reorganization and cross-linking. Exposed hydrophilic amino acid residues form new intermolecular bonds with water molecules, enhancing water retention and hydration.

Microstructural observations showed that ultrasonic treatment resulted in smaller, denser pores, which corresponded to better water distribution and gel texture. Additionally, color analysis indicated reduced gel color intensity, attributed to pigment degradation caused by the cavitation effect, which also likely contributed to the improved water retention properties.

Ultrasonic rehydration significantly improves the rehydration performance of SPI/KGM dry gels, with optimal results observed at 675 W. This treatment enhances rehydration efficiency, water distribution, and gel porosity, addressing challenges such as prolonged rehydration time and uneven water distribution in protein-based dry gels. These findings provide a robust model reference and theoretical guidance for SPI gel rehydration.

Pore models with varying diameters and hydrophilicity were constructed to explore the rehydration mechanism of SPI/KGM dry gels. Simulation results demonstrated a negative correlation between pore size and rehydration rate, with smaller pores facilitating faster water absorption. This relationship can be attributed to the increased capillary action in smaller pores, which enhances water penetration. Coupled with experimental data, the simulations clarified water diffusion within the gel, offering valuable insights for optimizing rehydration performance in practical applications.

The safety and performance of SPI/KGM gels in food applications were also evaluated. While SPI is widely used in food, it poses risks for individuals with soy allergies, necessitating proper labeling. In contrast, KGM is considered safe and suitable for most consumers. During cooking, gel structure, hydration, and texture are influenced by temperature, time, and pH. Heat-induced swelling and hydration improve water retention but may slightly reduce elasticity.

Visual analysis revealed a slight yellow hue in the gel (positive b-value in CIELab measurements), which may affect the appearance of visually sensitive products like fruits, vegetables, or desserts but has minimal impact on meats or frozen foods. Ingredient blending may be required to optimize appearance for specific applications.

Advancements in simulation technologies, such as micro-computed tomography (Micro-CT), allow detailed analysis of gel structures and their relationship with rehydration properties. High-resolution 3D imaging and modeling can reveal pore structures and simulate key phenomena like air-liquid interface dynamics and water transport [51]. These tools deepen understanding of rehydration mechanisms and provide theoretical support for optimizing SPI/KGM gel preparation and performance.

## 5. Conclusions

This study demonstrated that ultrasonic treatment significantly enhances the hydration performance of SPI/KGM dry gels, with the best results observed at 675 W. The cavitation effect facilitated faster hydration and improved water retention, addressing challenges such as prolonged hydration time and uneven water distribution. Simulation results revealed a negative correlation between pore size and hydration rate, providing insights into the role of pore structure in optimizing hydration performance. The integration of simulation and experimental findings elucidated the mechanism of water penetration and diffusion within the gel matrix. Ultrasonic treatment also induced structural changes, with denser pore formation and a shift in protein secondary structure from β-sheets to α-helices, further enhancing hydration efficiency. These findings provide a theoretical foundation and practical guidance for optimizing the hydration performance of SPI/KGM dry gels. By combining ultrasonic technology with pore structure optimization, this study offers innovative approaches for improving hydration processes and developing efficient, sustainable hydration technologies for food and other applications.

## Figures and Tables

**Table 1:** Changes in water relaxation time T2 and peak area of SPI/KGM dry gels under different ultrasonic powers.

	T21	T22	T23	A21	A22	A23
0 W	2.2478 ± 0.1384 ^b^	44.4878 ± 3.2471 ^a^	333.1295 ± 13.2465 ^a^	2.0742 ± 0.3284 ^d^	772.4027 ± 15.7636 ^e^	66.4572 ± 7.5349 ^c^
525 W	2.2478 ± 0.174 ^b^	41.5040 ± 5.4638 ^ab^	333.1295 ± 12.8734 ^a^	2.6124 ± 0.2675 ^cd^	1120.9516 ± 18.5632 ^d^	117.6298 ± 8.2554 ^b^
600 W	2.2478 ± 0.2335 ^b^	38.7204 ± 4.2536 ^ab^	333.1295 ± 15.2143 ^a^	2.7166 ± 0.1976 ^c^	1375.8475 ± 19.2367 ^c^	142.8419 ± 10.2246 ^b^
675 W	2.2478 ± −0.1236 ^b^	33.7006 ± 3.9458 ^b^	333.1295 ± 14.7135 ^a^	3.1197 ± 0.2258 ^ab^	1627.3083 ± 18.2563 ^b^	137.5806 ± 7.1695 ^a^
750 W	2.5826 ± 0.1534 ^a^	44.4878 ± 4.2687 ^a^	310.7867 ± 14.4456 ^a^	3.4523 ± 0.1664 ^a^	957.4201 ± 17.7712 ^a^	108.9065 ± 7.5563 ^a^

Note: Different superscript letters (a, b, c, d, e) within the same column indicate significant differences between samples (*p* < 0.05).

**Table 2:** Effect of sonication on the color of SPI/KGM dry gels.

	L*	a*	b*
0 W	64.38 ± 0.36 d	4.64 ± 0.11 a	9.10 ± 0.24 a
525 W	65.94 ± 0.28 c	4.52 ± 0.18 ab	9.01 ± 0.35 a
600 W	67.64 ± 0.45 b	4.39 ± 0.09 ab	8.31 ± 0.46 b
675 W	69.14 ± 0.24 a	4.34 ± 0.17 b	8.29 ± 0.18 b
725 W	69.75 ± 0.37 a	4.27 ± 0.19 b	7.69 ± 0.52 b

Note: Different letters (a, b, c, d) within the same column indicate significant differences between samples (*p* < 0.05). The symbol * represents variations in color intensity, with larger values indicating lighter or darker shades.

**Table 3:** Effect of sonication on the pore size of SPI/KGM dry gels.

Sample	Main Pore Size
0 W	138.21 ± 9.14 a
675 W	83.33 ± 7.57 b
750 W	146.78 ± 10.21 a

Note: Different letters (a, b) within the same column indicate significant differences between samples (*p* < 0.05).

**Table 4:** The setting of R in the model.

Sample	Main Pore Size (μm)	Definition
0 W	138.21 ± 9.14 a	R1 = 69.11
675 W	83.33 ± 7.57 b	R2 = 41.67
750 W	146.78 ± 10.21 a	R3 = 73.39

Note: Different letters (a, b) within the same column indicate significant differences between samples (*p* < 0.05).

## Data Availability

Data is contained within the article.

## References

[1] King T., Cole M., Farber J.M., Eisenbrand G., Zabaras D., Fox E.M., Hill J.P. (2017). Food safety for food security: Relationship between global megatrends and developments in food safety. Trends Food Sci. Technol..

[2] Lee A., Bensaada S., Lamothe V., Lacoste M., Bennetau-Pelissero C. (2022). Endocrine disruptors on and in fruits and vegetables: Estimation of the potential exposure of the French population. Food Chem..

[3] Feng L., Wang H., Ma X., Peng H., Shan J. (2021). Modeling the current land suitability and future dynamics of global soybean cultivation under climate change scenarios. Field Crops Res..

[4] Chen N., Zhao M., Chassenieux C., Nicolai T. (2017). The effect of adding NaCl on thermal aggregation and gelation of soy protein isolate. Food Hydrocoll..

[5] Zheng L., Regenstein J.M., Zhou L., Wang Z. (2022). Soy protein isolates: A review of their composition, aggregation, and gelation. Compr. Rev. Food Sci. Food Saf..

[6] Tang C. (2019). Nanostructured soy proteins: Fabrication and applications as delivery systems for bioactives (a review). Food Hydrocoll..

[7] Zhang T., Dou W., Zhang X., Zhao Y., Zhang Y., Jiang L., Sui X. (2021). The development history and recent updates on soy protein-based meat alternatives. Trends Food Sci. Technol..

[8] Tang C. (2017). Emulsifying properties of soy proteins: A critical review with emphasis on the role of conformational flexibility. Crit. Rev. Food Sci. Nutr..

[9] Zhang Q., Wang C., Li B., Li L., Lin D., Chen H., Liu Y., Li S., Qin W., Liu J. (2018). Research progress in tofu processing: From raw materials to processing conditions. Crit. Rev. Food Sci. Nutr..

[10] Khan M.S., Gowda B.H.J., Nasir N., Wahab S., Pichika M.R., Sahebkar A., Kesharwani P. (2023). Advancements in dextran-based nanocarriers for treatment and imaging of breast cancer. Int. J. Pharm..

[11] Muhoza B., Qi B., Harindintwali J.D., Koko M.Y.F., Zhang Q., Li Y. (2022). Combined plant protein modification and complex coacervation as a sustainable strategy to produce coacervates encapsulating bioactives. Food Hydrocoll..

[12] Yang N., Huang M., Gao C., Hu J., Liu Y., Nishinari K. (2024). Preparation and drug release performance of different gelation type polysaccharide/β-lactoglobulin fiber composite gels. Int. J. Biol. Macromol..

[13] Muhoza B., Xia S., Wang X., Zhang X. (2020). The protection effect of trehalose on the multinuclear microcapsules based on gelatin and high methyl pectin coacervate during freeze-drying. Food Hydrocoll..

[14] Fang Y., Li L., Vreeker R., Yao X., Wang J., Ma Q., Jiang F., Phillips G.O. (2011). Rehydration of dried alginate gel beads: Effect of the presence of gelatin and gum arabic. Carbohydr. Polym..

[15] Cassanelli M., Prosapio V., Norton I., Mills T. (2018). Acidified/basified gellan gum gels: The role of the structure in drying/rehydration mechanisms. Food Hydrocoll..

[16] Chien K.B., Aguado B.A., Bryc P.J., Shah B.N. (2013). In vivo acute and humoral response to three-dimensional porous soy protein scaffolds. Acta Biomater..

[17] Silva R., Bulut B., Roether J.A., Kaschta J., Schubert D.W., Boccaccini A.R. (2014). Sonochemical processing and characterization of composite materials based on soy protein and alginate containing micron-sized bioactive glass particles. J. Mol. Struct..

[18] Ran X., Yang H. (2022). Promoted strain-hardening and crystallinity of a soy protein-konjac glucomannan complex gel by konjac glucomannan. Food Hydrocoll..

[19] Wang Y., Zhang H., Liang Q., Guo X., Niu Z., Qiu S., Xu W., Li R. (2024). Effect of konjac glucomannan with different degrees of deacetylation on the gel behavior of transglutaminase induced soybean protein isolate emulsion gels. Food Hydrocoll..

[20] Cui T., Chen C., Jia A., Li D., Shi Y., Zhang M., Bai X., Liu X., Liu C. (2021). Characterization and human microfold cell assay of fish oil microcapsules: Effect of spray drying and freeze-drying using konjac glucomannan (KGM)-soybean protein isolate (SPI) as wall materials. J. Funct. Foods.

[21] Li C., Fan X., Sun Y., Zhou C., Pan D. (2022). Preparation, morphology and release of goose liver oil microcapsules. Foods.

[22] Xu S., Zhou Z., Liu Z., Sharma P. (2023). Concurrent stiffening and softening in hydrogels under dehydration. Sci. Adv..

[23] Fauster T., Giancaterino M., Pittia P., Jaeger H. (2020). Effect of pulsed electric field pretreatment on shrinkage, rehydration capacity and texture of freeze-dried plant materials. LWT.

[24] Salehi F., Goharpour K., Kamran H.R. (2023). Effects of ultrasound and microwave pretreatments of carrot slices before drying on the color indexes and drying rate. Ultrason. Sonochem..

[25] Wang R., Li Y., Sun G., Wang C., Liang Y., Hua D., Chen L., Mo H. (2023). Effects of Rehydration Time, Temperature and Ultrasound Treatment on Rehydration, Quality and Structure Properties of Low Moisture Texturized Soybean Protein. Food Biophys..

[26] Giancaterino M., Werl C., Jaeger H. (2024). Evaluation of the quality and stability of freeze-dried fruits and vegetables pre-treated by pulsed electric fields (PEF). LWT.

[27] Subrahmanyam K., Gul K., Paridala S., Sehrawat R., More K.S., Dwivedi M., Jaddu S. (2024). Effect of cold plasma pretreatment on drying kinetics and quality attributes of apple slices in Refractance window drying. Innov. Food Sci. Emerg. Technol..

[28] Zhou Y., Vidyarthi S.K., Zhong C., Zheng Z., An Y., Wang J., Wei Q., Xiao H. (2020). Cold plasma enhances drying and color, rehydration ratio and polyphenols of wolfberry via microstructure and ultrastructure alteration. LWT.

[29] Li Y., Wang S., Zhang G., Liu X., Liu H., He Y., Zhu D. (2022). Morphological and structural changes in thermally-induced soybean protein isolate xerogels modulated by soybean polysaccharide concentration. Food Hydrocoll..

[30] Wang X., Su Y., Wang Y., Chen X., Chen X., Liu Z. (2023). The Effect of Ultrasound on the Rehydration Characteristics of Semi-Dried Salted *Apostichopus japonicus*. Foods.

[31] Maeda Y., Hayashi H., Fukushima J., Takizawa H. (2021). Sonochemical effect and pore structure tuning of silica xerogel by ultrasonic irradiation of semi-solid hydrogel. Ultrason. Sonochem..

[32] Harnkarnsujarit N., Kawai K., Watanabe M., Suzuki T. (2016). Effects of freezing on microstructure and rehydration properties of freeze-dried soybean curd. J. Food Eng..

[33] Benseddik A., Azzi A., Zidoune M.N., Khanniche R., Besombes C. (2019). Empirical and diffusion models of rehydration process of differently dried pumpkin slices. J. Saudi Soc. Agric. Sci..

[34] Peng J., Lu L., Zhu K., Guo X., Chen y., Zhou h. (2022). Effect of rehydration on textural properties, oral behavior, kinetics and water state of textured wheat gluten. Food Chem..

[35] Vásquez U., Siche R., Miano A.C. (2021). Ultrasound-assisted hydration with sodium bicarbonate solution enhances hydration-cooking of pigeon pea. LWT.

[36] Lopez Quiroga E., Prosapio V., Fryer P.J., Norton I.T., Bakalis S. (2020). Model discrimination for drying and rehydration kinetics of freeze-dried tomatoes. J. Food Process Eng..

[37] Zhang X., Guo Q., Shi W. (2023). Ultrasound-assisted processing: Changes in gel properties, water-holding capacity, and protein aggregation of low-salt Hypophthalmichthys molitrix surimi by soy protein isolate. Ultrason. Sonochem..

[38] Zhang M., Li J., Su Y., Chang C., Li X., Yang Y., Gu L. (2019). Preparation and characterization of hen egg proteins-soybean protein isolate composite gels. Food Hydrocoll..

[39] Bao G., Niu J., Li S., Zhang L., Luo L. (2022). Effects of ultrasound pretreatment on the quality, nutrients and volatile compounds of dry-cured yak meat. Ultrason. Sonochem..

[40] Miano A.C., Ibarz A., Augusto P.E.D. (2016). Mechanisms for improving mass transfer in food with ultrasound technology: Describing the phenomena in two model cases. Ultrason. Sonochem..

[41] Pashazadeh H., Zannou O., Koca I. (2020). Modeling of drying and rehydration kinetics of *Rosa pimpinellifolia* fruits: Toward formulation and optimization of a new tea with high antioxidant properties. J. Food Process Eng..

[42] Sun Q., Yu X., Zhang L., Yagoub A.E.A., Tang Y., Wahia H., Zhou C. (2022). Effects of vacuum ultrasonic infiltration and combined drying on rehydration quality of ginger (*Zingiber officinale Roscoe*). Ind. Crops Prod..

[43] Kumar N., Kachhadiya S., Nayi P. (2020). Storage stability and characterization of biochemical, rehydration and colour characteristics of dehydrated sweet corn kernels. J. Stored Prod. Res..

[44] Soysal F., İsmail O. (2017). Investigation of the effect of temperature and pretreatment on the rehydration capacities of dried nectarine slices. Acta Sci. Technol..

[45] Patero T., Augusto P.E. (2015). Ultrasound (US) enhances the hydration of sorghum (*Sorghum bicolor*) grains. Ultrason. Sonochem..

[46] Gao X., Xie Y., Yin T., Hu Y., You J., Xiong S., Liu R. (2021). Effect of high intensity ultrasound on gelation properties of silver carp surimi with different salt contents. Ultrason. Sonochem..

[47] Mauerer A., Lee G. (2006). Changes in the amide I FT-IR bands of poly-l-lysine on spray-drying from α-helix, β-sheet or random coil conformations. Eur. J. Pharm. Biopharm..

[48] Liu F., Zou H., Hu J., Liu H., Peng J., Chen Y., Lu F., Huo Y. (2016). Fast removal of methylene blue from aqueous solution using porous soy protein isolate based composite beads. Chem. Eng. J..

[49] Nowacka M., Wedzik M. (2016). Effect of ultrasound treatment on microstructure, colour and carotenoid content in fresh and dried carrot tissue. Appl. Acoust..

[50] Flores-López S.L., Karakashov B., Ramírez-Montoya L.A., Menéndez J.A., Fierro V., Arenillas A., Montes-Morán M.A., Celzard A. (2021). Effect of the porosity and microstructure on the mechanical properties of organic xerogels. J. Mater. Sci..

[51] Wang F., Du D., Chen H., Zhang C. (2019). Simulation of evolution mechanism of dynamic interface of aqueous foam in narrow space base on level set method. Colloids Surf. A Physicochem. Eng. Asp..

